# Hierarchical representation of shapes in visual cortex—from localized features to figural shape segregation

**DOI:** 10.3389/fncom.2014.00093

**Published:** 2014-08-11

**Authors:** Stephan Tschechne, Heiko Neumann

**Affiliations:** Faculty of Engineering and Computer Science (with Psychology and Education), Institute of Neural Information Processing, Ulm UniversityUlm, Germany

**Keywords:** ventral pathway, distributed representation, figure-ground segregation, modulatory feedback, computational model

## Abstract

Visual structures in the environment are segmented into image regions and those combined to a representation of surfaces and prototypical objects. Such a perceptual organization is performed by complex neural mechanisms in the visual cortex of primates. Multiple mutually connected areas in the ventral cortical pathway receive visual input and extract local form features that are subsequently grouped into increasingly complex, more meaningful image elements. Such a distributed network of processing must be capable to make accessible highly articulated changes in shape boundary as well as very subtle curvature changes that contribute to the perception of an object. We propose a recurrent computational network architecture that utilizes hierarchical distributed representations of shape features to encode surface and object boundary over different scales of resolution. Our model makes use of neural mechanisms that model the processing capabilities of early and intermediate stages in visual cortex, namely areas V1–V4 and IT. We suggest that multiple specialized component representations interact by feedforward hierarchical processing that is combined with feedback signals driven by representations generated at higher stages. Based on this, global configurational as well as local information is made available to distinguish changes in the object's contour. Once the outline of a shape has been established, contextual contour configurations are used to assign border ownership directions and thus achieve segregation of figure and ground. The model, thus, proposes how separate mechanisms contribute to distributed hierarchical cortical shape representation and combine with processes of figure-ground segregation. Our model is probed with a selection of stimuli to illustrate processing results at different processing stages. We especially highlight how modulatory feedback connections contribute to the processing of visual input at various stages in the processing hierarchy.

## 1. Introduction

We visually perceive our environment as a stable and comprehensive combination of objects, where we can easily identify objects and persons and we efficently analyse geometrical cues that allow a precise navigation and interaction. This happens so effortlessy and accurately that it is absolutely counterintuitive that this is an extraordinary achievement of our brain. The visual system of mammals achieves this result from input that is captured at the retinal level after light has been projected through the eye and hits light-sensitive neurons. The perception of our environment starts at this local level where our position, the direction of our gaze, the current illumination, an object's surface properties and its location relative to others causes a set of neurons in the retina to respond with increased activation that is a function of received light intensity. How the visual system transforms this concert of local visual inputs into a stable and informative perception of surfaces and objects is subject to intense research. Since the pioneering works on neural principles by Hubel and Wiesel (1959) many insights into cortical processing of visual input has been discovered. Neurophysiologists agree that the processing in the mammalian brain is performed in a hierarchical way and processing is organized into various specialized brain areas (Felleman and Van Essen, 1991). Those brain areas receive connections from preceding processing stages, but also from regions later in the processing stream (Markov et al., 2013). Early areas in visual cortex are retinotopically arranged (Hubel and Wiesel, 1962), which means that juxtaposed retinal locations are mapped to juxtaposed locations in visual cortex, with foveal positions being represented at a higher resolution. Individual assemblies of neurons become activated when their preferred stimulus is presented in their receptive field (Hubel and Wiesel, 1962). With progression in the visual pathway, the size of those RFs increase from sizes smaller than one degree of visual angle to sizes covering a good part of the visual field. In parallel, the tuning toward the preferred stimulus changes from simple features like oriented contrasts (Hubel and Wiesel, 1962) to complex ones like image features, figure-ground-related cues, object categories or faces. Processing along the visual pathway is organized into two streams (Ungerleider and Haxby, 1994), the ventral stream that exhibits a tuning toward movement and position, whereas the dorsal stream processes shapes and objects.

However, most of the achievements that the visual system exhibits, like the abilities to generalize and its robustness and adaptability, most probably stem from connections that connect higher cortical areas with lower ones (Hupé et al., 1998; Markov et al., 2013). Those feedback connections are believed to play an important role in visual processing, as they enrich local activations with contextual information that is represented at higher visual areas. We propose that on the way from generalizing early local features to higher meaningful representations, the role of object boundaries plays an essential part. Contrasts indicate spatial changes in local illumination which might coincide with object boundaries that allow segregation from background. However, contrasts indicating a real transition from one object to another or from the object to the background must be separated from those indicating an illumination change and those caused by textured regions. This must be accomplished using contextual information. The region delimited by such a boundary is a surface with locally constant parameters, and a set of surfaces forms objects, scenes and eventually our complete visual environment. We believe that the processing capabilities of early and intermediate stages of visual cortex are used to transform local representation into an intermediate, more meaningful representation of contours, shapes and surfaces. Following those ideas, we propose that a stable representation of shape may be established by interacting assemblies that are each devoted to specific features properties. We thus propose a hierarchical model of 2-dimensional shape representation that incorporates processing at low and intermediate areas of visual cortex. Each model area consists of a three-stage processing cascade of initial filtering, application of modulatory feedback effects and center-surround interactions leading to an activity normalization (Carandini and Heeger, 1994; Carandini et al., 1999; Kouh and Poggio, 2008; Carandini and Heeger, 2012). The functional effects of this columnar cascade can roughly be mapped onto compartments of cortical area subdivisions [as suggested in (Self et al., 2012)].

Our model combines the representation of visual shapes with mechanisms for figure-ground segregation on the basis of assigning border ownership and incorporates a distributed representation of local contour curvature over different cortical areas. In our model we emphasize the computational role of feedforward and feedback mechanisms (Grossberg, 1980; Edelman, 1993) to generate a hierarchical distributed representation of shape information. The feedback amplifies the sensory signal such that the subsequent competition between neurons builds a competitive advantage (Tsotsos, 1988; Girard and Bullier, 1989; Desimone, 1998; Roelfsema et al., 2002; Reynolds and Heeger, 2009). Boundaries and their orientation are represented after intial processing in model area V1 and a grouping stage in model area V2. Contextual boundary configurations are also represented at a coarser spatial level at model V2 and V4 to achieve selectivities toward contour curvature. With the influence of feedback, those cells are enhanced at lower stages that contribute to a matching bottom-up signal.

The output of our model is a representation of shapes and shape segments where contextually compatible boundary information benefits from recurrent feedback connections. Such a representation could provide input to subsequent processing stages for e.g., object classification tasks, which would clearly benefit from the enhanced representation.

This model extends previous own works (Neumann and Mingolla, 2001; Hansen and Neumann, 2004; Weidenbacher and Neumann, 2009) but introduces functional properties that have been inspired by the works of other groups. A model of curvature representation can also be found in Cadieu et al. (2007). The authors modeled physiological findings of the same group (Pasupathy and Connor, 1999; Connor et al., 2007) that has focussed on the dynamics of contour processing (Yau et al., 2013). Cell representations from early visual areas are combined to intermediate-level shape descriptors are used in a computational model by Rodríguez-Sánchez and Tsotsos (2012). Riesenhuber and Poggio (1999, 2000); Mutch and Lowe (2008) released very powerful models of object and object class categorization in a hierarchical modeling approach. The physiological (Zhou et al., 2000; O'Herron and von der Heydt, 2011) as well as the computational (Layton et al., 2012) aspects of border ownership are subject to intense research. Models of contour integration and perceptual grouping also exist from Zhaoping (1998) and Jehee et al. (2006); Roelfsema (2006). The role of feedback and physiological investigations are elaborated in Hupé et al. (1998); Markov et al. (2013) and very recently (De Pasquale and Murray Sherman, 2013) found evidence for the modulatory properties of feedback in the visual cortices of mice.

## 2. Model definition

**Figure 1:**
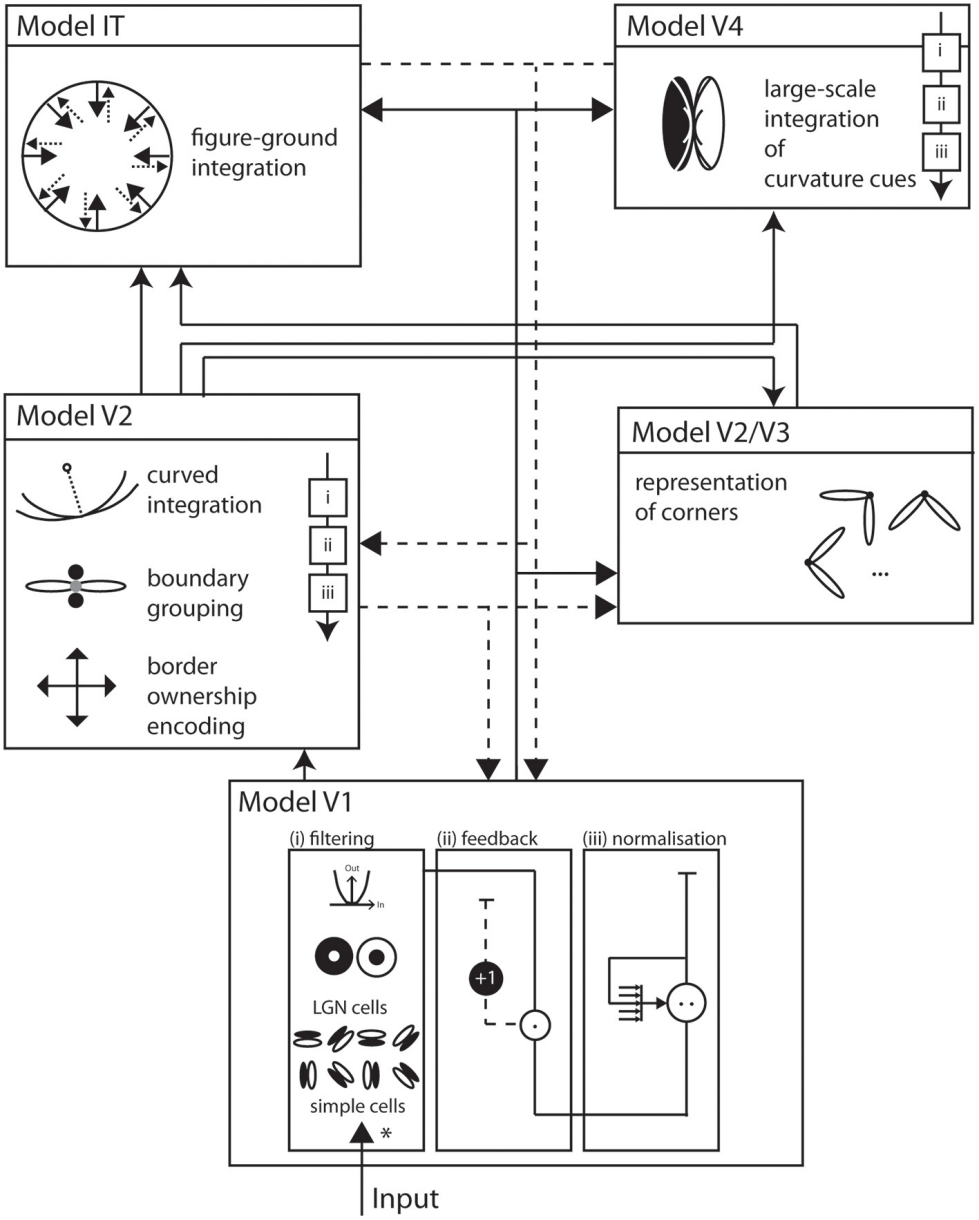
**Overall model architecture**. Visual input enters the model at the bottom and is subsequently processed by interconnected functional areas with increasingly large receptive field sizes. Solid arrows indicate feedforward, dashed arrows indicate feedback, or modulatory, connections. Each area implements a generic architecture of building blocks that consists of (i) filtering (*) of the input, (ii) modulation by feedback, and (iii) response normalization. *Model V1* consists of image filters that resemble properties of early processing in LGN and V1, namely simple and complex cells that are tuned to circular or elongated image contrasts. *Model V2* integrates responses of model V1 with long-range integration cells. A multiplicative combination of subcells responds best to elongated contrasts of one dominant orientation. Also at V2, a population of cells represents border ownership directions. At population of long-range curved integration cells help represent different boundary curvatures. The *Models V2, V3* complex hosts representations of corners by integrating V1 responses from orthogonal configurations over a small spatial surround. *Model V4* consists of cells that asymmetrically integrate responses from V1 and V2 to become curvature selective at an increased spatial scale. In *Model IT*, cells with large receptive fields integrate responses from V1, V2 and V4 at local figure convexities to achieve a contextual segregation into figure and ground. Area V4 allows a description of a shape by means of cues that are represented on distributed areas in the model. Those cues exist at different spatial scales and their mutual interaction generates dynamic processes in the model.

We propose a biologically inspired model of two-dimensional shape representation that consists of a hierarchical structure of interconnected model areas (see Figure 1). These model areas resemble the mechanisms of early and intermediate stages of visual processing in the ventral pathway of visual cortex. Each of the model areas is represented by a staged columnar cascaded model (see Figure 1). This cascade consists of (i) initial filtering, (ii) activity modulation, and (iii) center-surround interaction.

### 2.1. Nomenclature

The following list familiarizes the reader with the nomenclature that is used in our manuscript:

- Names of model areas are written in superscripts to indicate the affiliation to parameters or responses. A response for cells in area V1 thus would read *R*^*V*1^.
- Greek symbols (like α, β, σ) are used for parameters of dynamic functions or shapes of receptive fields.
- N and M are constants indicated the number of orientations and directions used in our model.
- An instance of an orientation is indicated using the step width $\hat{\theta }$ between discretely sampled orientations. The variable *i* is used as index. A specific orientation in a population is indicatd with *i*$\hat{\theta }$ with *i* ∈ 0..*N* − 1.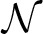 stands for a normal (Gaussian) distribution that can be isotropic or anisotropic, rotated and spatially shifted, as defined by subscripted parameters.
- Spatial positions are denoted in bold latin letters like **x** or **p**.
- To indicate an angle between two vectors we use ∡(**v**_1_,**v**_2_).
- The convolution operation is abbreviated using the asterix (*).
- A rectification operation is indicated by ⌈...⌉^+^.

### 2.2. Processing cascade

In our model, neural activations or response levels are modeled using a scalar representation of the neural firing rate. For ease of writing, we will in the following refer to the response of *a cell*, keeping in mind that this represents the activation level of a large number of real cells. The first model stage of the cascade is the initial filtering of available input *I*. To model the response for the preferred stimulus in the visual field, we employ a 2-dimensional convolution operation with the preferred stimulus as the convolution kernel *K_pref_*. The response of model cells *R* = *I* * *K_pref_* is defined as

(1) $$R(x,y)=\sum_{u=-\infty }^{\infty }\sum_{v=-\infty }^{\infty }K_{pref}(u,v)I(x-u,y-v)\;\forall x,y\in D_{I}$$

A frequently used kernel in our model serves as elementary building block and is a 2-dimensional Gaussian distribution that is elongated along one axis and rotated around its center. We refer to this distribution by 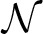 with parameters for orientation θ, deviation along the axes σ_1_, σ_2_ and the center of the distribution **μ**.

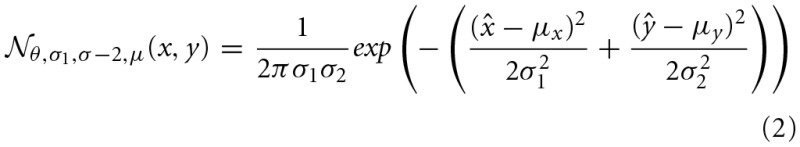



with

(3) $$\left(\begin{array}{c} \hat{x}\\ \hat{y} \end{array}\right)=\left(\begin{array}{c} x\\ y \end{array}\right)\left(\begin{array}{c} cos\;\theta \;-sin\;\theta \\ sin\;\theta \;cos\;\theta \end{array}\right)$$

If parameters are not specified they are considered having the following default values: θ = 0, **μ** = (0,0)*^T^*, σ_1_ = 1, σ_2_ = σ_1_. In the following, functional filter kernels will often be designed as a combination of multiple such elementary components.

The coefficients of the kernel that models the preferred stimulus might incorporate negative weights to account for the inhibitory connections a cell may receive. This could lead to overall responses that are numerically negative. We thus use a rectification operator after convolution and feedback stages to ensure that numerically the response rate of a population is not negative:

(4) $${\left\lceil R\right\rceil }^{+}=max(0,R).$$

**Figure 2:**
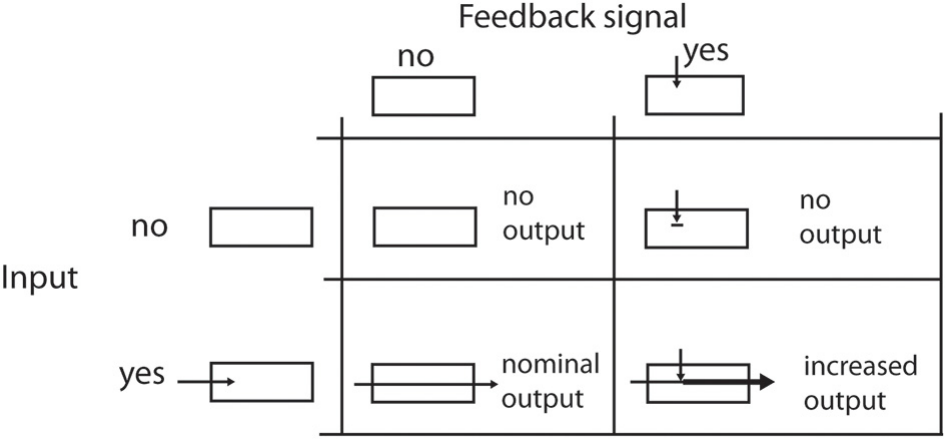
**Effects of feedback on the signal flow**. The four table cells illustrate the effects when a feedback signal and/or an input signal is available. Please note that a feedback signal alone cannot elicit any cell response in the modeled area. It only enhances the response level when the filtering of the input signal generates some output.

At the second stage of the cascade, response levels are modulated by recurring input from higher visual areas. We propose a feedback mechanism that excerpts a purely modulatory gain control on the input. That means that feedback alone cannot generate activities without activation by the initial filtering step (see Figure 2). With *R* being the unmodulated driving signal and *net_FB_* being the strength of the feedback, the modulated response is

(5) $$R_{FB}\propto R\cdot (1+net_{FB}).$$

Using this approach, given *R* = 0 no signal is generated as output irresponsible of the strength of the feedback *net_FB_*. On the other hand, if no feedback signal is available, the right part of the equation leaves the input signal *R* unchanged (Salin and Bullier, 1995; Hupé et al., 1998; Eckhorn, 1999; Gilbert and Li, 2013).

Before normalization at the final stage of the cascade, we apply a non-linear transfer function to map the computed responses to a cell activation level. In our model, we use a function of type

(6) $$f(R)=R^{k}$$

with *k* the non-linearity parameter. At the final stage, we incorporate a mechanism that keeps the response level limited by using a *shunting inhibition* that leads to a non-linear compression of high amplitude activities resembling the *Weber-Fechner-Law* of perceptual thresholds. In its dynamic formulation, the rate of change of the signal ∂*_t_R^norm^*_θ_ depends on the current activation level as well as the amount of input *I_net_*:

(7) $$\partial_{t}R_{i\hat{\theta }}^{norm}=-\alpha R_{i\hat{\theta }}^{norm}+\beta R_{i\hat{\theta }}-R_{i\hat{\theta }}^{norm}\cdot I_{net}$$

(8) $$I_{net}=\frac{1}{N}\sum_{i=0}^{N-1}R_{i\hat{\theta }}.$$

With *N* the size of the used population, respective orientations. When this equation is solved at equilibrium, i.e., when ∂*_t_R*_*i*$\hat{\theta }$_ = 0, the activation becomes

(9) $$R_{i\hat{\theta }}^{norm}=\beta \frac{R_{i\hat{\theta }}}{\alpha +I_{net}}$$

The constants influence the steepness of the non-linearity (α) and the scale of the normalized signal (β). This model architecture has previously been used in various approaches touching different domains, such as the disambiguation of local motion (Bayerl and Neumann, 2004; Beck and Neumann, 2011), the processing of transparent motion (Raudies and Neumann, 2010) the detection of texture boundaries (Thielscher and Neumann, 2003), the extraction of object boundaries using texture compression (Weidenbacher and Neumann, 2009), and the analysis and representation of biological motion sequences (Layher et al., 2014).

In the following, we describe the forward sweep of our model, from early toward intermediate processing stages. After all areas have been described in detail, we will elaborate on the feedback connections that build the recurrent model structure.

### 2.3. Model area V1

The processing starts at early stages of visual cortex where we model the functionality of LGN and V1 cells where LGN cell responses provide feedforward input to V1 cells. Here, the visual input is intially processed to generate a representation of local image contrasts and local contrast orientations (Hubel and Wiesel, 1962).

In general, model cell responses follow first-order dynamics and represent the changes of membrane potentials. Such dynamics are influenced by excitatory and inhibitory inputs and a passive decay of activity. In order to simplify the computations in our large-scale simulations we use steady-state equations in calculations of feedforward filtering stages. Others are numerically integrated using a Euler one-step scheme. The response of LGN cells is calculated using the following linear equation,

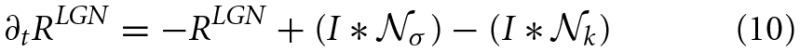



As pointed above, we assume that such linear feedforward filtering operations quickly relax at their equilibrium state. Therefore, we utilize the steady-state equation

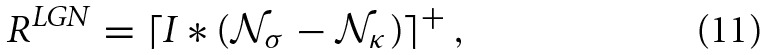



with σ and κ denoting the width of center and surround kernel, respectively. To model cells that are tuned to oriented contrasts, we use elongated gaussian kernels that are combined into odd-symmetric simple cell profiles using anisotropic σ_1_ and σ_2_ and a radius ω_1_ for the spatial shift of the integration kernels. The responses of such cells are denoted by the steady-state equation

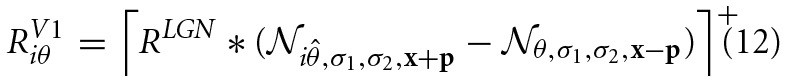



with

(13) $$p=\omega_{1}{\left(cos(\theta +\pi ),sin(\theta +\pi )\right)}^{T}$$

The filter kernel that is defined that way yields high response activations at positions with local luminance contrasts that match the layout of the filter kernel. To achieve insensitivity against the sign of contrast, pairs of equally oriented filters with opposite sensitivity to contrast polarity are used. Such filters populate a set with evenly distributed orientation tunings that represent possible contrast orientations. The locally dominant orientation can be derived by selecting the orientation channel with maximum response, *i_max_* = *argmax_i_R*^*V*1^_*i*$\hat{\theta }$_.

### 2.4. Model area V2/V3 complex

At the stage of V2 we model cells sensitive to contextual influences of contour segments that are arranged in larger spatial extent compared to V1 receptive fields. The integration of elongated contours in V2 makes use of a mechanism that links cells of like orientations over larger spatial distances. The filters are modeled using elongated Gaussian kernels positioned at **p** with offset ω^*V*2^_*ex*_ to the center of the cell. The parameters of the elongated Gaussian kernels are set to build a combined kernel of an elongated integration field, which reflects the highly significant anisotropies of long-range connections in visual cortex (Bosking et al., 1997). The subfields sample the activations generated by V1 complex cells (Grossberg and Mingolla, 1985; Neumann and Sepp, 1999).

The subfields are combined in a multiplicatively. This resembles a logical *and*-operation for the individual subfield activations. Modeled V2 cells only become activated when both subfields receive sufficient input. The response is thus able to bridge local gaps in contours. This is in line with physiological findings, as V2 neurons respond to elongated luminance contrasts as well as to illusory contours (von der Heydt et al., 1984; Heitger et al., 1998) like in the Kanisza square.

This integration mechanism is enhanced by local inhibitory effects. Smaller and isotropic integration fields are positioned along an orthogonal axis from the cell's center with distance ω^*V*2^_*inh*_, building a cross-like zone of excitatory and inhibitory integration, compare (Piëch et al., 2013). At those positions **p**⊥, activity from all orientations is integrated and has an inhibitory effect on the total response. This has a strong suppressive effect on contour fragments that are positioned within a cluttered surround, while isolated boundary segments are not affected. The complete response for an elongated V2 cell is calculated by the steady state equation:

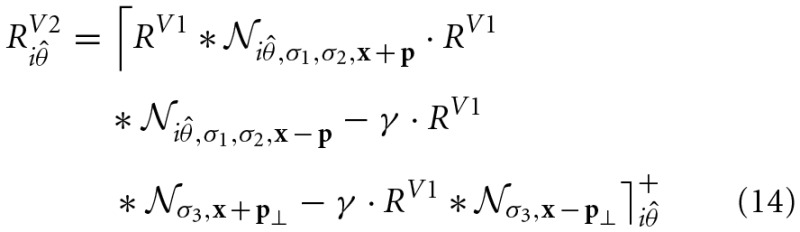



with

(15) $$p=\omega_{ex}^{V2}{\left(cos(i\hat{\theta }),sin(i\hat{\theta })\right)}^{T}$$

(16) $$p_{⊥}=\omega_{inh}^{V2}{\left(cos(i\hat{\theta }+\pi ),sin(i\hat{\theta }+\pi )\right)}^{T}$$

We also model V2 neurons that respond to more complex stimuli like in curved or angular shape outlines. We propose a population of V2 cells tuned to curved contour outlines that allows integration of smooth and even fragmented boundary configurations (Field et al., 1993). We propose a population of V2 cells tuned to a curved contour outline, see **Figure 4**. They resemble the functionality of elongated V2 cells but their integration fields are designed such that they are curved. A curvature direction is defined either to the left or the right of the tangent orientation at the target location. the center of curvature defines an osculating circle with given curvature-radius. The integration weight is modeled by a function *w^dist^* that decreases with distance from the cell's center. A second tuning function *w^ori^* in the orientation domain specifies the weights for the orientation population. Here, the weight decreases with distance to the main tuning direction which is perpendicular to the dominant orientation. Basically, only those orientations are integrated with maximum that are tangential to the curvature trace at their relative positions. This yields a sharp tuning of the cell for a certain curvature level. The complete response for an curved V2 cell is calculated by the steady state equation:

(17) $$R_{i\hat{\theta }}^{V2Curv}=\sum_{x}w(\omega^{C},x)\cdot R_{x,i\hat{\theta }}^{V1}\text{ with}$$

(18) $$w=w^{dist}\cdot w^{ori}$$

(19) $$w^{dist}=exp(-\frac{{\left(x-x_{0}\right)}^{2}}{\sigma_{1}^{2}})$$

(20) $$w^{ori}=sin(∡(\vec{x_{0}x},\vec{xc}))\cdot exp\left(-\frac{{\left(\|\vec{xc}\|-\omega^{c}\right)}^{2}}{\sigma_{2}^{2}}\right)$$

(21) $$c=x_{0}+\omega^{c}{\left(cos(i\hat{\theta }),sin(i\hat{\theta })\right)}^{T}$$

In this equation *w* denotes a weighting function for responses in the currently integrated position **x**. The reference point of the integrating cell is **x_0_**. The weighting functions depends of the curvature radius being integrated *w^c^*. *w^dist^* produces a weight depending on the distance from the curvature cell's center **x_0_**. *w^ori^* returns a weight given the current angle between integrating position and center of curvature **x_c_**, depending on the reference position *x*_0_. In simple words, orientations orthogonal to the imaginary line between integration position and imaginary curvature center **c** receive highest weight. *w^ori^* is extended with a function that drops with increased distance of integrating position to imaginary center of curvature.

Cells in visual cortex V2 also show selectivity to the figure-ground arrangement of the scene in the visual field (Williford and von der Heydt, 2013). So-called border ownership cell responses are elicited when figure of arbitrary shape is presented on their preferred side with respect to the center of their receptive field. From the same group, O'Herron and von der Heydt (2013) have also shown that during visual motion caused by eye motion or object motion, these border ownership signals are remapped to different neurons. The visual system uses this information to resolve depth arrangements in the stimulus (Qiu and von der Heydt, 2005). The pointing of border ownership cells indicates the direction of the frontal surface at every image location. This reflects to commonly known Gestalt rule that a boundary is owned by the frontal figure.

We model border ownership cells by a retinotopically arranged population representing four potential directions where the figure can be positioned relative to the cell's center. Border ownership responses are initially isotropic and only occur together with local contrast activations. Cells indicating opponent border ownership direction are mutually rivaling in our model. The complete response for an border ownership V2 cell is calculated by the steady state equation:

(22) $$R_{\lambda }^{V2Bown}=\{\begin{array}{l} \begin{array}{lll} f(R_{i\hat{\theta }}^{V2}) & \text{when} & \lambda ⊥i\hat{\theta }\\ 0 & \text{when} & \lambda ∥i\hat{\theta } \end{array} \end{array}$$

The mutual competition between activations indicating opposing border ownership directions *R^Bown^_a_* and *R^Bown^_b_* is calculated by

(23) $$\partial tR_{a}^{Bown}=-\alpha \cdot R_{a}^{Bown}+A(1-R_{a}^{Bown})-\beta \cdot R_{b}^{Bown}$$

(24) $$\partial tR_{b}^{Bown}=-\alpha \cdot R_{b}^{Bown}+A(1-R_{b}^{Bown})-\beta \cdot R_{a}^{Bown}$$

Based on empirical evidence of neural representations generated by cells selective to multiple orientations (Felleman and Van Essen, 1987; Ito and Komatsu, 2004; Anzai et al., 2007) we incorporate model representations of corners in a dedicated model area V2/V3 complex. We build upon the proposal developed in Weidenbacher and Neumann (2009) that corner and junction configurations can be made explicit by specific read-out mechanisms. Here, we employ a simplified version as of Hansen and Neumann (2004) to generate corner representations by grouping V1 responses of orthogonal orientation fields. In a steady-state formalism the response reads

(25) $$R_{i\hat{\theta }}^{V2/V3}={\left\lceil R_{i\hat{\theta }}^{V1}\cdot R_{i\hat{\theta }+\pi }^{V1}\right\rceil }^{+}$$

### 2.5. Model area V4

Inspired by experimental evidence cells in model V4 integrate responses of V1,V2, and V2/V3 to achieve a selectivity that considers large-scale boundary fragments as well as local variations in curvature and a selectivity for corners (Pasupathy and Connor, 1999; Yau et al., 2013). Curvature selective cells are modeled in a two-stage cascade of mechanisms. The first level integrates V2 contour responses and is selective to curvature directions, left or right (relative to the cell's orientation preference). The second level combines opposite curvature directions into one response, like in V1 complex cells. This model mechanism differs from the one proposed by Rodríguez-Sánchez and Tsotsos (2012). which utilizes single stage filter computations. In this approach specific subfield mechanisms sensitive to orientation, tangential contour outline and scale are combined in a non-linear fashion to selectively respond to contour fragments of different curvatures. We develop a mechanism that is distributed over different stages to first group responses to extended contour outlines in V1 and V2 suppressing non-contour clutter. In the case of sharply localized corners and junctions the dedicated representations of localized multi-orientation responses will be activated. Those responses of grouping cells (or the junction representations) are integrated at the subsequent stage. Here, curvature selectivity is made explicit that distinguishes left and right curvatures. Different integration scales generate selectivity to curvature. This distribution allows to associate regions of high contour curvature at an intermediate scale with localized outline details at the finer scale which enhances the selectivity of the model developed by Rodríguez-Sánchez and Tsotsos (2012).

The model cell responses in our model are described by the following equations:

(26) $$\begin{array}{l} \partial_{t}R_{i\hat{\theta }}^{V4,left}=-\alpha_{4}R_{i\hat{\theta }}^{V4,left}+\left(1-R_{i\hat{\theta }}^{V4,left}\right)\cdot A_{i\hat{\theta }}\\ \;-\;\left(1+R_{i\hat{\theta }}^{V4,left}\right)\cdot B_{i\hat{\theta }} \end{array}$$

(27) $$\begin{array}{l} \partial_{t}R_{i\hat{\theta }}^{V4,right}=-\alpha_{4}R_{i\hat{\theta }}^{V4,right}+\left(1-R_{i\hat{\theta }}^{V4,right}\right)\cdot A_{i\hat{\theta }}\\ \;-\;\left(1+R_{i\hat{\theta }}^{V4,right}\right)\cdot B_{i\hat{\theta }} \end{array}$$

with

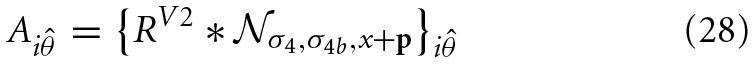

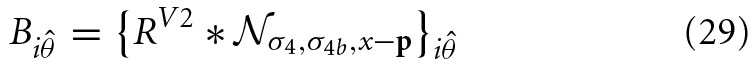





(30) $$p=\omega^{V4}{\left(cos(i\hat{\theta }),sin(i\hat{\theta })\right)}^{T}$$

These responses are calculated at equilibrium and averaged subsequently, leading to the model V4 filter response

(31) $$R_{i\hat{\theta }}^{V4}=\frac{1}{2}\frac{{\left\lceil \left(A_{i\hat{\theta }}-B_{i\hat{\theta }}\right)\right\rceil }^{+}+{\left\lceil B_{i\hat{\theta }}-A_{i\hat{\theta }}\right\rceil }^{+}}{\alpha_{4}+A_{i\hat{\theta }}+B_{i\hat{\theta }}}$$

This integration mechanism yields a response for locally curved boundary segments at a larger spatial scale. For elongated contour segments that show no curvature, the response of individual cells will be equal and the combined response very low.

### 2.6. Model area IT

So far, we have described how our model integrated local features from model V1 into elongated, potentially curved boundaries at model V2–V4. Model area IT performs contextual integration that allows a segregation into figure and ground and a representation of prototypical objects at a large spatial scale. As discussed above, a population of V2 cells responds selectively to the direction of figure-ground direction. The local representation of border ownership at model V2 represents a set of available local hypotheses that cannot locally be resolved, as this step requires contextual influence from a larger spatial surround. Cells in IT cortex have been shown to be shape selective with properties generalizing over contrast polarity and mirror reversal (Baylis and Driver, 2001). The authors demonstrate that such cells do not, however, generalize over the assignments of figure-ground direction. The investigation supports the view that the population of probed IT cells is mainly driven by the sidedness of contours and less so by the contour itself. Given the rapidness of ownership selectivity observed in V2, we propose that ownership computation relies on a network of V2–V4–IT cell interaction. Our model uses local shape configuration in the outline of an object to collect confidence about the direction of figure and ground. We adopt an approach of Zhou et al. (2000) and model an integration cell at model IT that integrates border-ownership hypotheses from a larger spatial extent from model V2 input. For each location in the image, border ownership activations in a local neighborhood that point toward the inside of the respective receptive field contribute to the activation of an IT cell. This results in strong responses in model IT where local image regions are surrounded by contour convexities. Local activities of border ownership cells in model V2 then receive a positive enhancement if they contributed to such an integration process. This recurrent architecture resolves the initially ambiguous assignment of border ownership. Taken together, this makes the model belong to the class of feedback architectures according to the categorization in Williford and von der Heydt (2013). The response of cells and their interaction is denoted by the following equations:

(32) $$\begin{array}{l} R_{x_{0}}^{IT}=\sum_{i}^{N-1}\sum_{p}f(x_{0},p,i\hat{\theta })\cdot R_{i\hat{\theta },x_{0}+p}^{V2}\\ \;\cdot exp\left(-\frac{{\left(\omega_{IT}-\|\vec{x_{o};x_{0}+p}\|\right)}^{2}}{\sigma_{IT}^{2}}\right) \end{array}$$

with

(33) $$f=cos\left(∡\left(\vec{x_{0};x_{0}+p},i\hat{\theta }\right)\right)$$

Such an IT cell at position **x_0_** integrates responses of V2 cells in its proximity **p**. The integration weight *f* depends on the angle between **x_0_** and **x_0_ + p** and the currently integrated orientation *i*$\hat{\theta }$. This grants orientations parallel to an imaginary line toward *x*_0_ high weights, while orthogonal orientations receive low weights.

This model area receives connections from the early as well as from the intermediate functional stages V1 and V2 where curvature is represented. This means that high-resolution local cues as well as contextual cues like corners from a larger region are available. A shape can thus be described as a set of contributing prototypical elements that contribute to the local configuration at every image location. Those elements are not solely generated through integration of lower areas, but exist as a distributed representation in all modeled areas and profit from mutual interaction through feedback and exhibit dynamic processes when a stimulus is presented.

### 2.7. Feedback for contour enhancement

The mechanisms so far presented contributed to the feedforward sweep of the model. We stated earlier that in visual cortex (and in neural processing in general), the input of cortical areas of higher stages highly contribute to the performance of individual earlier areas. By such recurring connections, contextual information is introduced in lower regions. We are thus now going to focus on the recurrent connections that are incorporated in our model.

Let's briefly recall that we model feedback connections that have a modulatory effect (Girard and Bullier, 1989) as outlined in Section 2, Equation 5. In Figure 2 we illustrate how a feedback signal alone cannot elicit responses as long as no input activation is present. On the other hand, feedback that matches input configurations will increase those activations. We stick to this convention throughout our following elaborations.

V2 long-range and curved cells represent continuous straight or curved contours. Their multiplicative combination of receptive field subcomponents caused the cells to elicit responses whenever a contour of matching orientation was presented in their receptive fields. Now, those cells in V1 that contributed to the integration process will receive feedback and be thus increased in activity. The following non-linear transformation stage increases the difference in response strength with respect to other oriented contour cells that did not receive feedback. At the subsequent normalization stage, local response levels are now slightly increased by the recurrent input. Now, surrounding activations without feedback have a competitive disadvantage and receive a higher divisive normalization relative to their activation due to the increase response in their neighborhood that contributed to the sum. The dynamics of these interactions are denoted in formal terms. The enhancement of filter responses (Equation 12) via modulating feedback is defined by

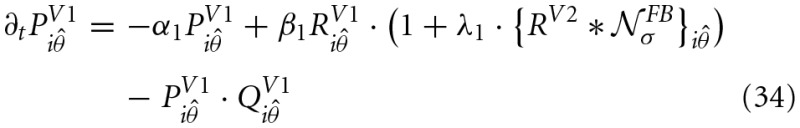



**Figure 3:**
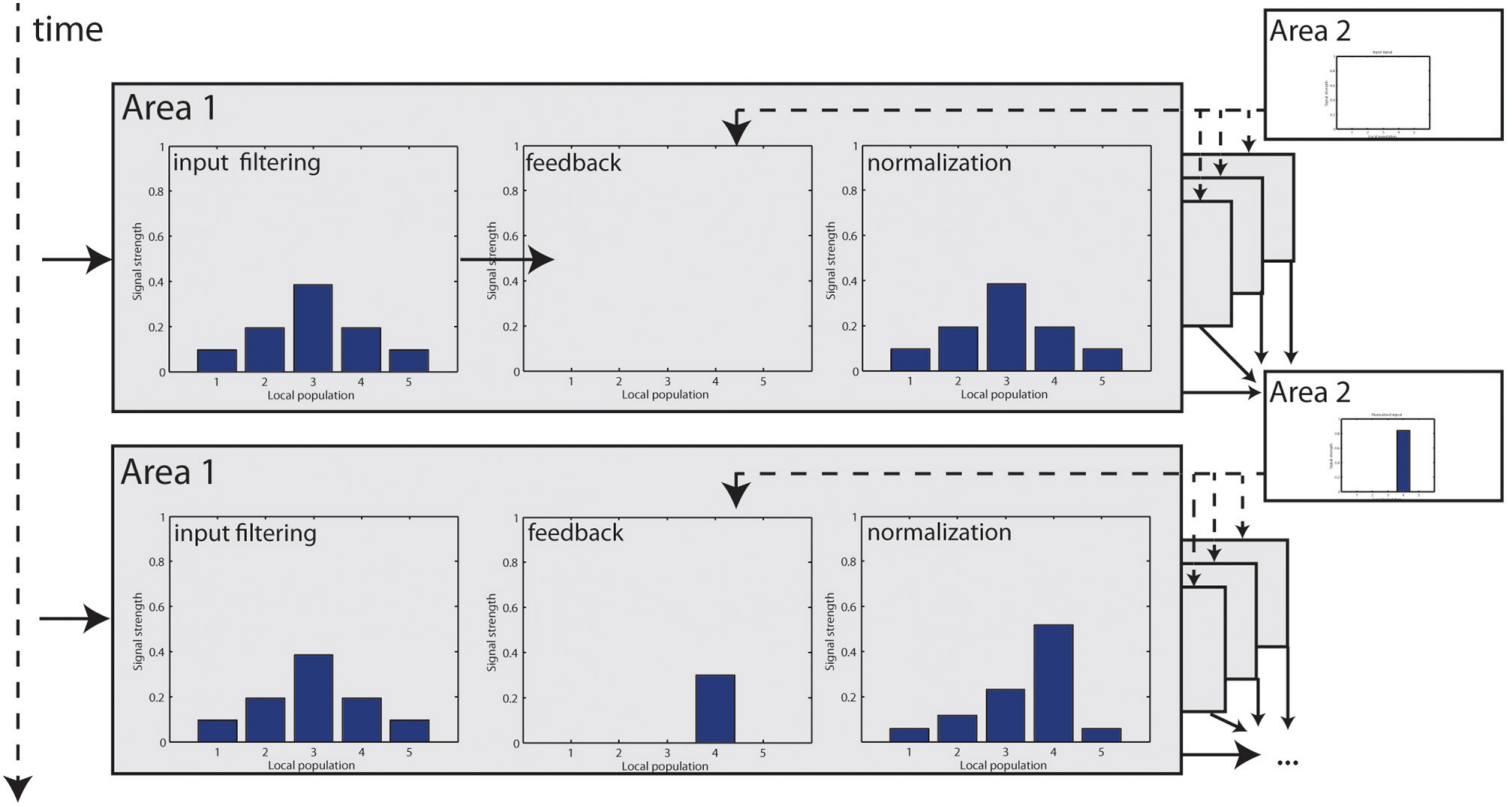
**Step-by-step illustration of how feedback modulation is dynamically incorporated in the model**. At *t* = 1, visual input is filtered and elicits responses in *Area 1*. Initially, no feedback signal is available which leaves the signal unchanged. Responses are finally normalized. Those responses now become integrated at *Area 2* to elongated contours and a feedback signal is generated, which enters *Area 1* at *t* = 2. Now, some responses are accentuated, resulting in a higher cumulated response level that is used for normalization (which is further intensified by a stage of non-linear transformation). Unmodulated responses are damped in relation to *t* = 0.

The subsequent competition to accomplish activity normalization is defined as

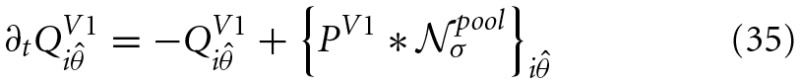



with TODO parameters. Figure 3 shows an illustrated version of the mechanism with a small numerical model.

**Figure 4:**
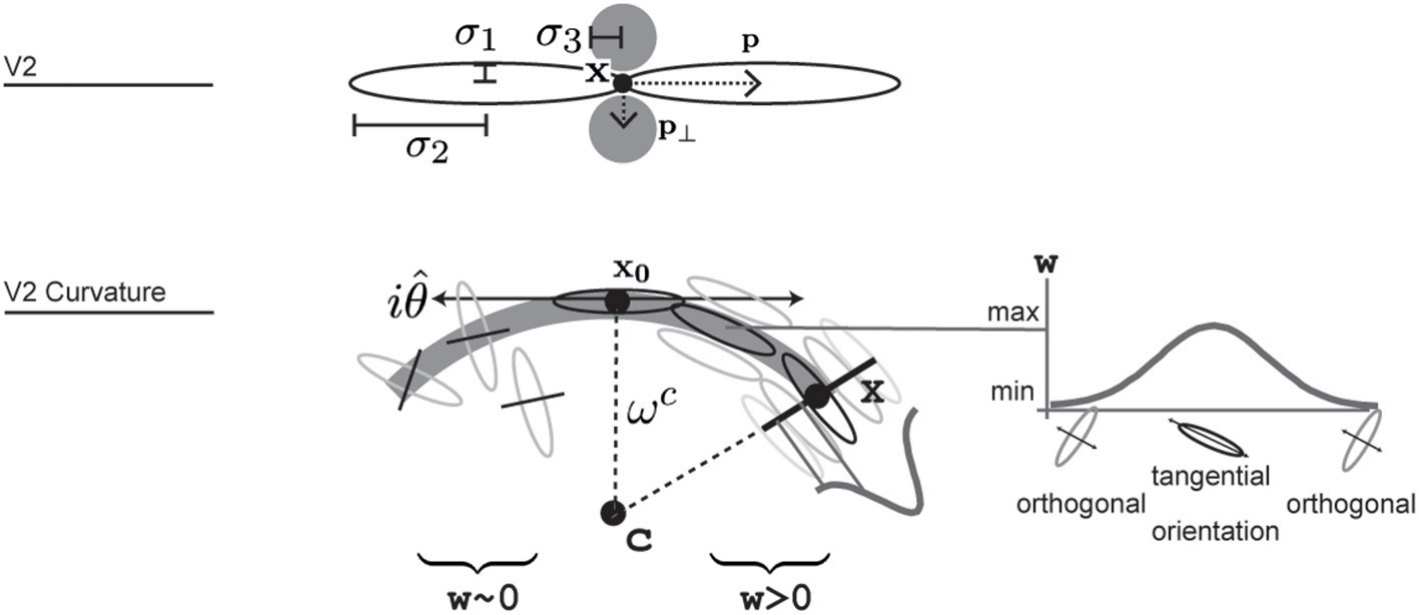
**Illustration of how V2 long range and V2 curvature cells are designed and where parameters are used**. V2 cells integrate from two larger excitatory and two smaller inhibitory regions. V2 curvature cells integrate responses of oriented cell along a curvilinear path that forms around an imaginary central point **c**. Integration weights additionally depend on the distance from the cell's reference point **x_0_**, angular difference to the tangential trajectory and a function of local radius, indicated here by a gaussian profile. Correctly aligned orientations that result in a large integration weight are shown in the right side of the arc, while some that result in weights close to zero are shown along the left arc in this illustration.

### 2.8. Feedback for curvature representation

As stated earlier, the modeled V4 cell do not at all or only marginally respond to straight elongated contours. Responses of V2 cells to curved boundaries are integrated in model V4, where integration cells sensitive to opposite sign of curvature mutually compete for equal orientations. These cells respond at positions with a local curved contour configuration, but are silent at elongated straight contours. Feedback is generated for those V2 cells that contribute to those curved boundary segments the corresponding model V4 cells respond to maximally. Regions with curved boundary segments thus elicit a strong response of V4 cells while regions with mostly straight contours do not elicit such a strong response. This signal can thus be used to differentiate regions of many straight contour segments from regions with many curved contours.

In formal terms, the V2–V4 cell interactions are defined by

(36) $$R^{V2curv}=-\alpha R_{i\hat{\theta }}^{V2curv}+(1-R_{i\hat{\theta }}^{V2curv})\cdot A(1+R_{i\hat{\theta }})\;$$

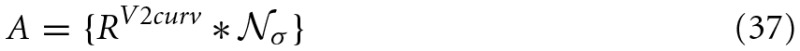

### 2.9. Feedback for figure-ground segregation

The contribution of feedback to figure-ground segregation is twofold in our model. First, local hypotheses of border ownership are generated by intra-area recurrent connections from long-range grouping cells. Contextual feedback from model IT resolves the remaining ambiguities. Initially, all directions of border ownership are equally likely at boundaries. With increasing confidence about local contrast orientations generated by V1 and V2, two options for border ownership directions are discarded and only two orthogonal border ownership directions remain. Activations of long-range V2 cells that indicate elongated surface boundaries and their orientation locally increase activities of those border ownership cells that are directed perpendicularly to the orientation of the boundary. Activity normalization for V2 border ownership cells then leads to a suppression of activities for ownership directions orthogonal to the boundary orientation. Formally, this is accomplished by the dynamics

(38) $$\begin{array}{l} \partial_{t}R_{i\hat{\phi }}^{BOwn}=-R_{i\hat{\phi }}^{BOwn}+\beta (R_{i\hat{\theta }}^{V2}+h_{tonic})\\ \;-\;R_{i\hat{\phi }}^{BOwn}\cdot \sum_{\gamma }R_{i\hat{\phi }}^{BOwn} \end{array}$$

with

(39) $$\theta =\phi +\frac{1}{2}\;mod\;\pi .$$

Second, V2 border ownership cells receive feedback from cells in model IT. Here, border ownership as well as figural cues, e.g., from local junctions, or curvature maxima, were integrated by IT cells. For the correct inference of figure and ground, feedback from IT to V2 is essential. Figure-Ground cells at IT level integrate border ownership activations from V2 in a circular fashion to integrate the coherence of directions indicating a convex pattern of figure outline. In the feedback sweep, this contextual information is now fed back to these border ownership cells compatible with the configuration using recurrent connections. In formal terms, this extends the dynamics presented in Equation 38 above by incorporating a modulating feedback signal from model IT cells, namely

(40) $$\begin{array}{l} \partial_{t}R_{i\hat{\theta }}^{BOwn}=-R_{i\hat{\theta }}^{BOwn}+\beta (R_{i\hat{\theta }}^{V2}+h_{tonic})\cdot (1+\lambda_{2}\cdot R_{i\hat{\theta }}^{IT})\\ \;-\;R_{i\hat{\theta }}^{BOwn}\cdot \sum_{\gamma }R_{i\hat{\theta }}^{BOwn} \end{array}$$

This also concludes the feedback sweep of our recurrent model. In the following section, we will show the performance of the model and its individual areas in the Results Section.

## 3. Results

In this section we illustrate the capabilities of our model in a number of simulations. To demonstrate how the model processes shapes, we use some artificial images to show working principles of various subcomponents of our model. These simple shapes were taken from the *Webdings* font freely available with a Microsoft® Windows™ 8.1 operating system. We also include also a depiction of a *Kanisza* square (Kanizsa, 1955). This is a special stimulus because it elicits the perception of illusory contours at the outline of the occluding square, a sensation our model is also capable to represent.

To demonstrate the abilities of our model to process real world images we acquires the dataset of Fowlkes et al. (2007) and selected a few examples that we included in our Results Sections. These images have a resolution of 321 × 481 pixels in landscape or portrait orientation. They were converted to grayscale images using the Mathworks® Matlab® rgb2gray function which performs a perceptionally weighted combination of the red, green and blue channel. We used 8–12 iteration steps to allow recurrent feedback signals to build up. The angular resolution of cell populations is defined by selecting eight $\frac{\pi }{8}$ steps to encode orientation. Border ownership is represented by a population representing 4 directions. Model V2 curvature cells also used 8 orientations for tangential orientations, but due to two possible curvature directions, our model contains a population of 16 curvature cells. A list of parameters used is given in Table 1.

**Table 1:** **General model parameters used for simulations**.

**Description**	**See equation**	**Value**
Number of orientations used		8
Number of feedback iterations		6
Number of BOwn directions		4
**MODEL AREA V1**		
Network size		321 × 481
LGN σ	10	1.00
LGN κ		1.50
LGN normalization α	(9 applied)	2.17e-03
LGN normalization β	(9 applied)	2.17e-03
V1 contrast σ_1_	12	0.23
V1 contrast σ_2_		0.12
for **p**: Excentricity ω_1_		3.00
V1 normalization α	(9 applied)	2.16e-06
Non-linearity of V1 responses	(6 applied)	4.00
**MODEL AREA V2/V3**		
V1:V2 subsampling	14	1: 3
Network size		107 × 161
Filter size of V2 complex		41.00
V2 complex cell σ_1_, σ_2_, σ_3_		0.21,0.02,0.10
in **p**: ω^*V*2^_*ex*_		3.70
in **p**: ω^*V*2^_*inh*_		2.50
V2 inhibition strength γ		0.10
V2 nonlinearity *k*	(6 applied)	1.00
Strength of V2–V1 feedback		0.11
Non-linearity of BOwn		1.00
In **c**: curvature radius ω*^c^*	19	15.00
in *w^dist^*: σ_1_		40.00
in *w_ori_*: σ_2_		2.00
Strength of *R*^*V*2*Curv*^ feedback		0.15
**MODEL AREA V4**		
V1:V4 subsampling		1: 4
Network size		81 × 121
V4 filter size		31.00
α_4_	26	0.01
σ_4_,σ_4b_	28	0.43,1.35
ω^V4^	28	−1.00
**MODEL AREA IT**		
σ_IT_	32	0.43
σ		29.00
ω_IT_		17.00
IT Non-linearity		3.00
α	(9 applied)	5.49e-05
Strength of BOwn feedback		50.00

### 3.1. Early processing stages

**Figure 5:**
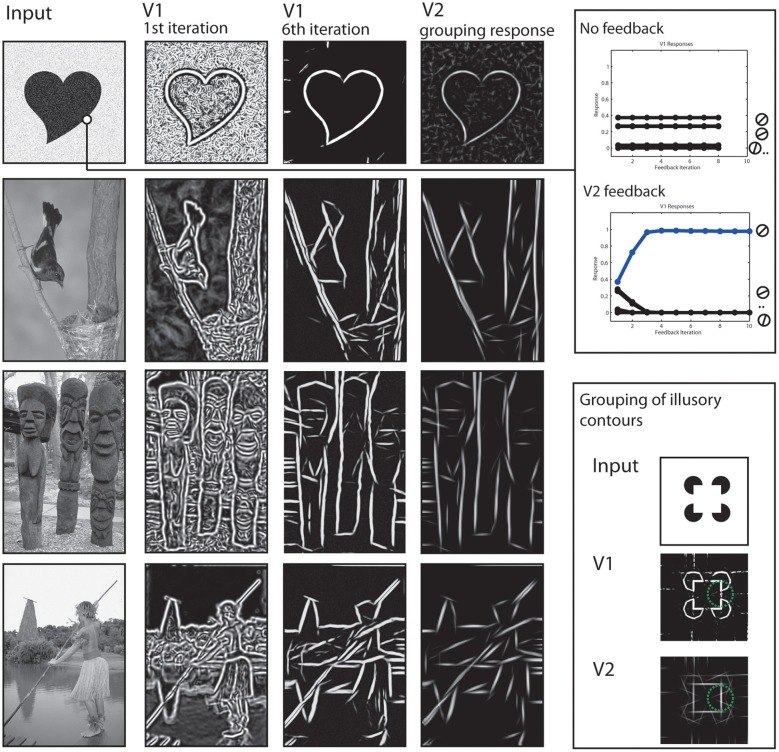
**Results of early processing stages V1 and V2**. Left column: Initial input images. Second and Third column: Cumulated responses of model V1 neurons at the initial processing iteration and a few iterations steps, respectively. Fourth column: Responses of model V2 neurons. Elongated edges formed by like-oriented contrasts are grouped as reflected by responses at respective locations. This stage also shows activations for illusory contours contours (third row) at the gaps between contrasts. Upper right box: The two plots indicate time courses for V1 activations. Initially, multiple V1 neurons are activated due to a broad tuning width (first plot). Without feedback, this effect prevails through iterations. With feedback, the correct orientation (blue) receives feedback and gradually reduces activations of other orientations (second plot). Lower right box: Example how model V2 neurons show responses at positions formed by illusory contours (in green circle) due to contextual integration.

To begin with, we show how the processing at early stages achieves a representation of the stimulus concerning contrasts and elongated contours. Local contrasts are represented in the early stages by model V1 and V2 cells. However, as can be seen in Figure 5 the responses rapidly change in the first few iteration steps. The contained contour as well as the added noise signal both elicit responses at the V1 level (*second column*) and cause the shapes outline to be not clearly separated from the background. However, those responses are grouped into elongated contour representations in model V2 (*4th column*). Elongated contour segments are clearly emphasized. From these V2 activations, a recurrent feedback signal is generated that modulates V1 activations. After a few iterations, the representation at V1 dramatically changed, with the outline of the figure now clearly visible.

The effect of the feedback signal is also measurable in a quantitative way, see Figure 5, *right*. Along the boundary of an object we plotted the activation levels of the population of V1 neurons that represent the orientation. Initially, the neuron with preferred orientation responds best, but also those with orientation tunings close to the real contour (*first plot*). The situation changes when feedback is added (*second plot*). Now, representations of undesired orientations are attenuated and the activation of the cell representing the contextually valid orientation is highly increased.

**Figure 6:**
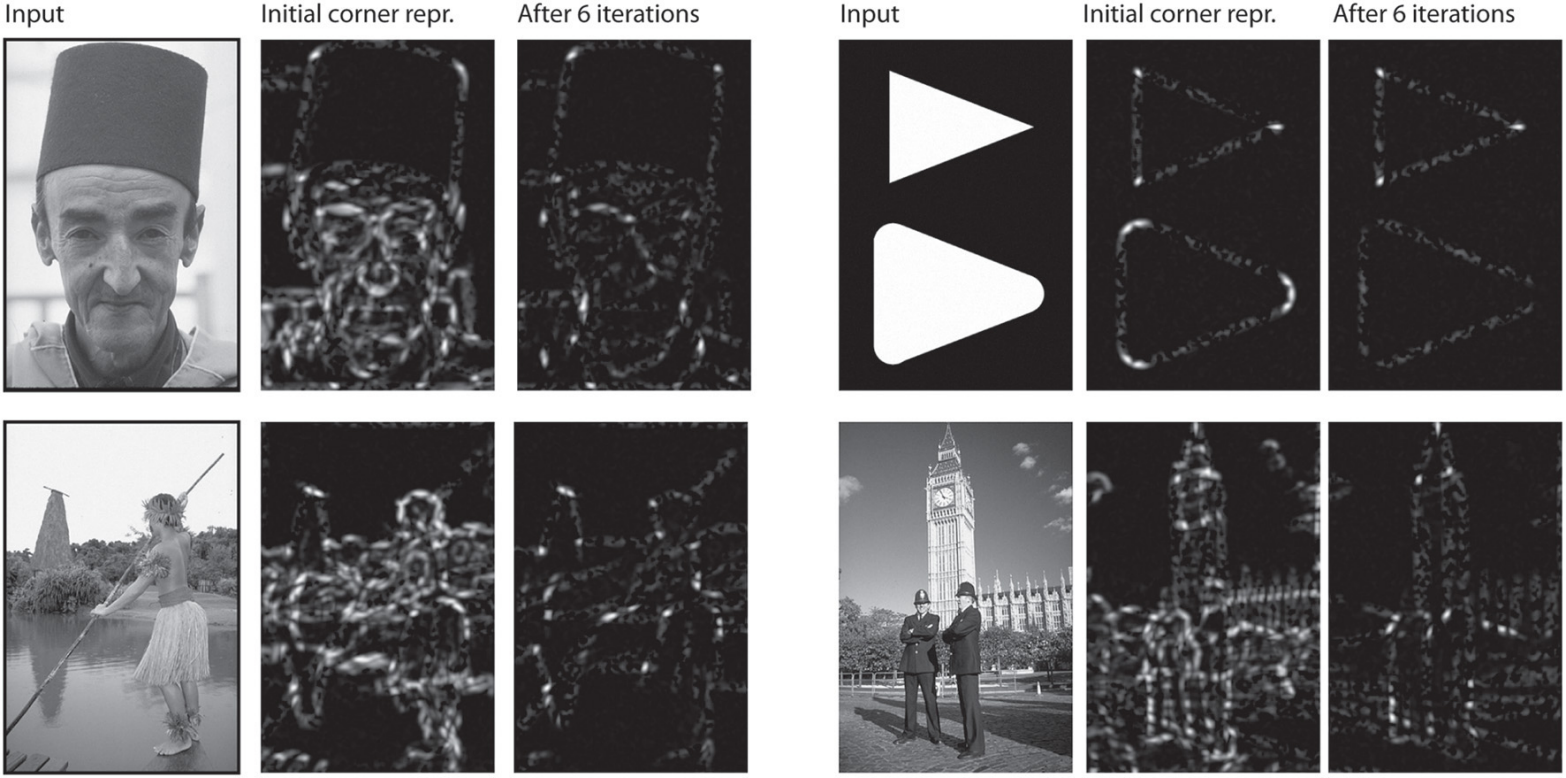
**Corner representation in model V2/V3**. For each group of three pictures: Initially, responses of model V1 did not yet benefit from contextual feedback of model V2 neurons. Corner representation is thus distorted by noise (second row, middle). After a few iterations, when V1 responses have been modulated V2 feedback, the corner representation is much clearer.

Also in Figure 5, the representation of illusory contours at V2 stage is depicted. This is illustrated using an input depicting a *Kanisza* square (*last row*). A complete square is highly salient for human observers despite the fact that only a series of circles with cut-out corners are depicted. This is reflected in the grouping responses of V2 neurons, they also show activity in the gap between the real contour fragments. Figure 5 shows V2 responses for the same parameter set and for a parameter set with changed receptive field sizes, to illustrate the effect even stronger (*framed part*). Figure 6 shows a result of the corner representation in the model.

### 3.2. Curvature tuning

**Figure 7:**
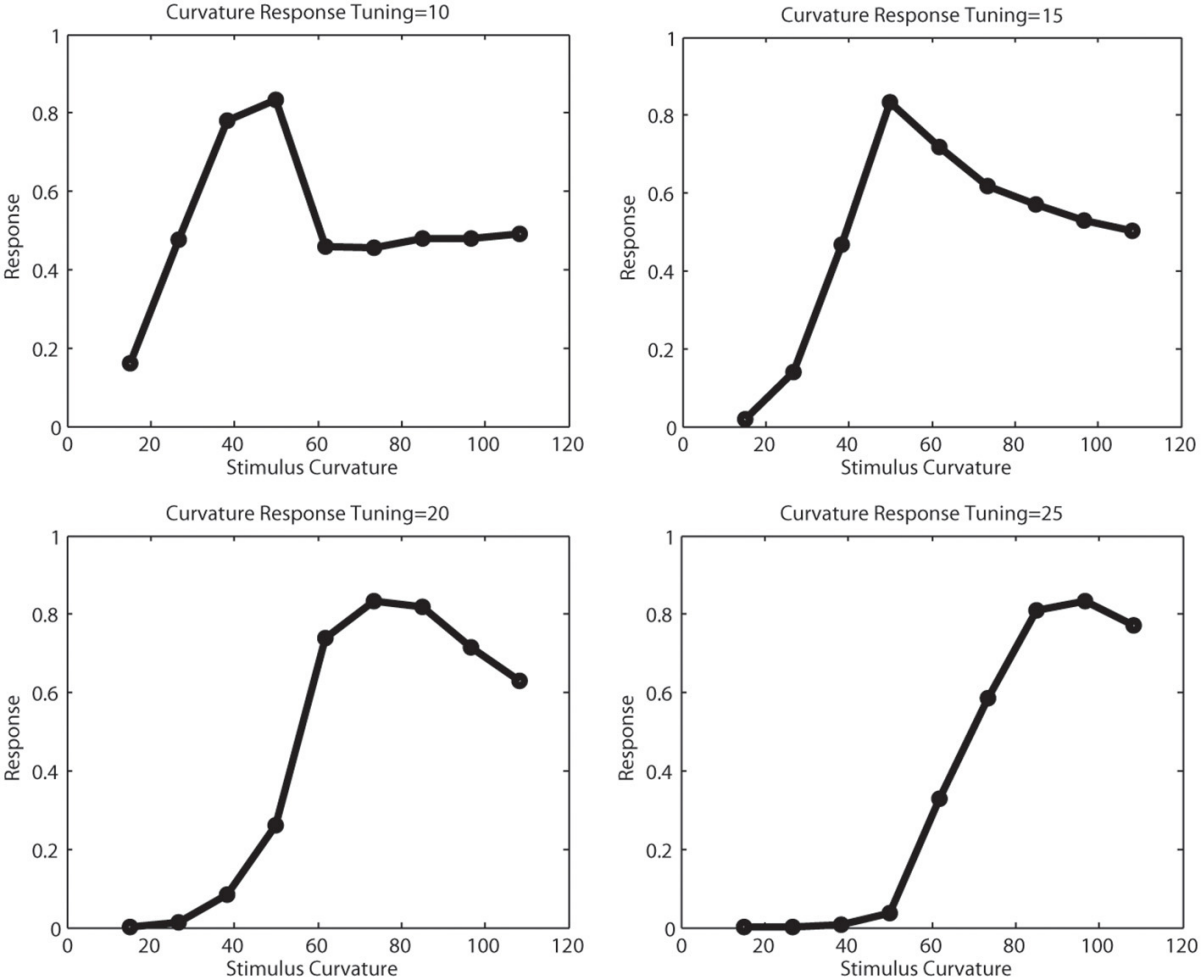
**Tunings of different curvature cells**. The x-axis show the curvature of the presented stimulus, the y-axis the response strength of a curvature cell tuned for 10,15,20, and 25 pixels curvature radius in model V2, which correlates for a curvature of 40,60,80, and 100 pixels in model V1. For the smaller curvature radii, subsampling artifacts cause the tuning function to be less smooth.

Figure 7 illustrates the tuning functions we defined for model V2 curved cells. A curved cell with distinct radius tuning was selected and we presented arcs of different curvature to this cell and simulated the response. We performed this for four cells with curvature tuning to 10, 15, 20, and 25 pixels radius. This curvature definition happens in V2, where the initial resolution of the image had been subsampled. For this reason, the value here correspond to values 40,60,80, and 100 in V1 resolution. In each plot, the peak response occured when the stimulus with the matching radius was presented. In this simulation, subsampling artifacts cause the first two plots to elicit some discontinuities.

### 3.3. Shape representation

**Figure 8:**
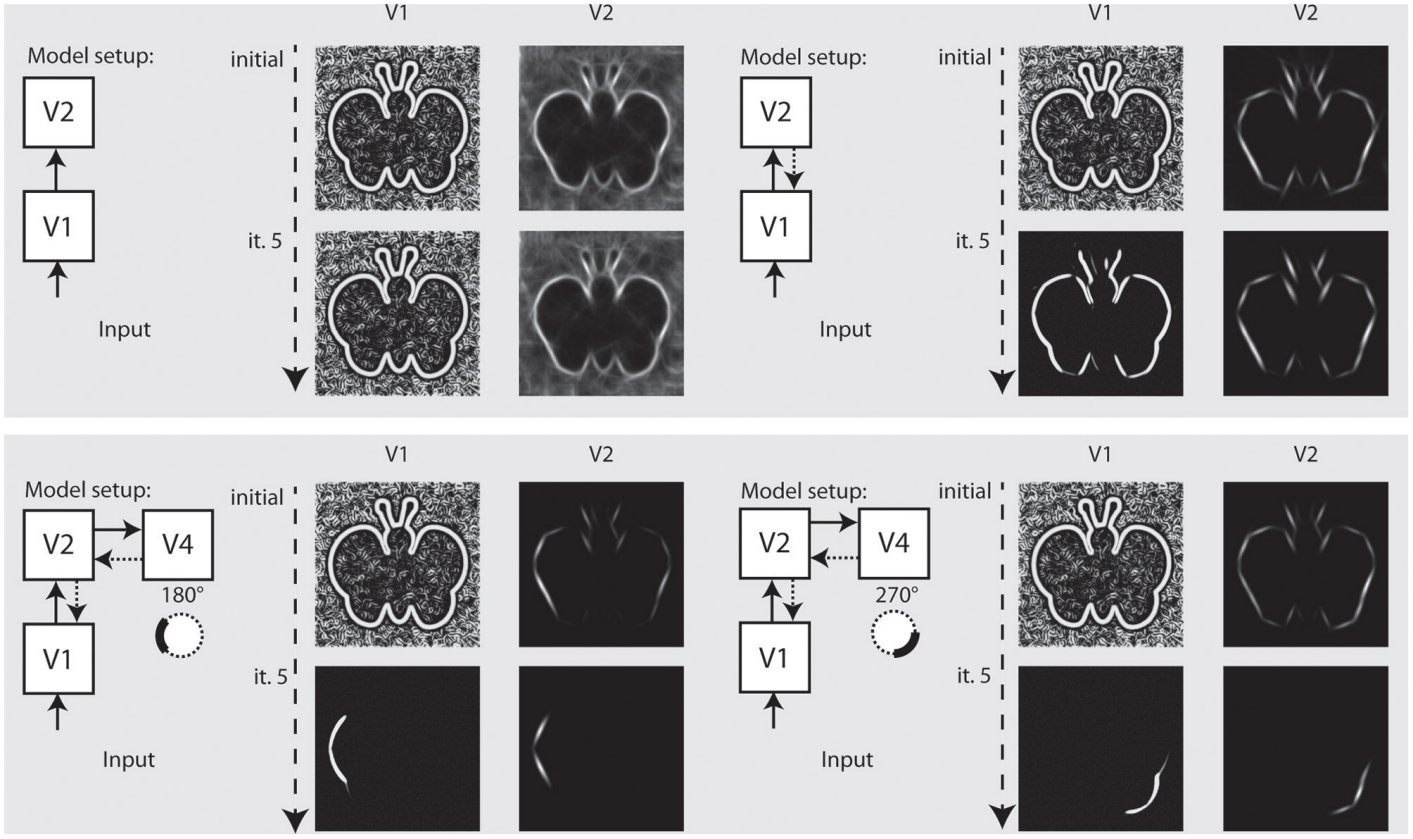
**Representation of curved boundary segments and effect of feedback**. Top left: Model setup without feedback connections. The initial representation at model V1 and V2 undergo no change. Top right: Feedback from V2 causes a refinement of elongated structure within a few iterations. Bottom left: Feedback from V4 allows the accentuation of boundary segments with distinct curvature strength and direction. Here, a curvature segment as found on the left part of the shape is highly emphasized by feedback. Bottom right: Same as left, but with selectivity for another segment of the shape. Note that while boundaries with the same orientation are present in the stimulus, only the one with matching curvature is emphasized.

In the final setup, we show how our model independently represents different elements of a shape, and how this depends on the recurrent feedback connections. Figure 8 illustrates the results we achieved for an artifical image. Initially, we configured the model to only use feedforward connections from V1 to V2. The model only achieves an representation at model V1 and a representation at V2 where the elongated boundaries are visible, but surrounded by many spurious activations. When recurrent feedback from V2 is added, the representation at V1 improves in the first few iterations before a steady representation is reached. In parallel, elongated boundaries at V2 are integrated and noise is highly reduced.

To represent prototypical objects at an intermediate level of detail, we stated that the model needs to represent different contour properties. In the second row of Figure 8 we show how the model achieves to emphasize V1 responses when they contribute to a certain contour fragment with desired properties. We deliberately exaggerated the effect and chose a very narrow tuning so that all other responses become almost completely suppressed. On the *left* side, we let the model emphasize contour parts that are oriented almost vertical but in a curved context of a matching radius. As can be seen, the model highlights that parts on the left side of the stimulus that matches and leaves others suppressed, even if their local orientation would match. On the *right* side of Figure 8, we perform the same for a different part of the shape outline.

**Figure 9:**
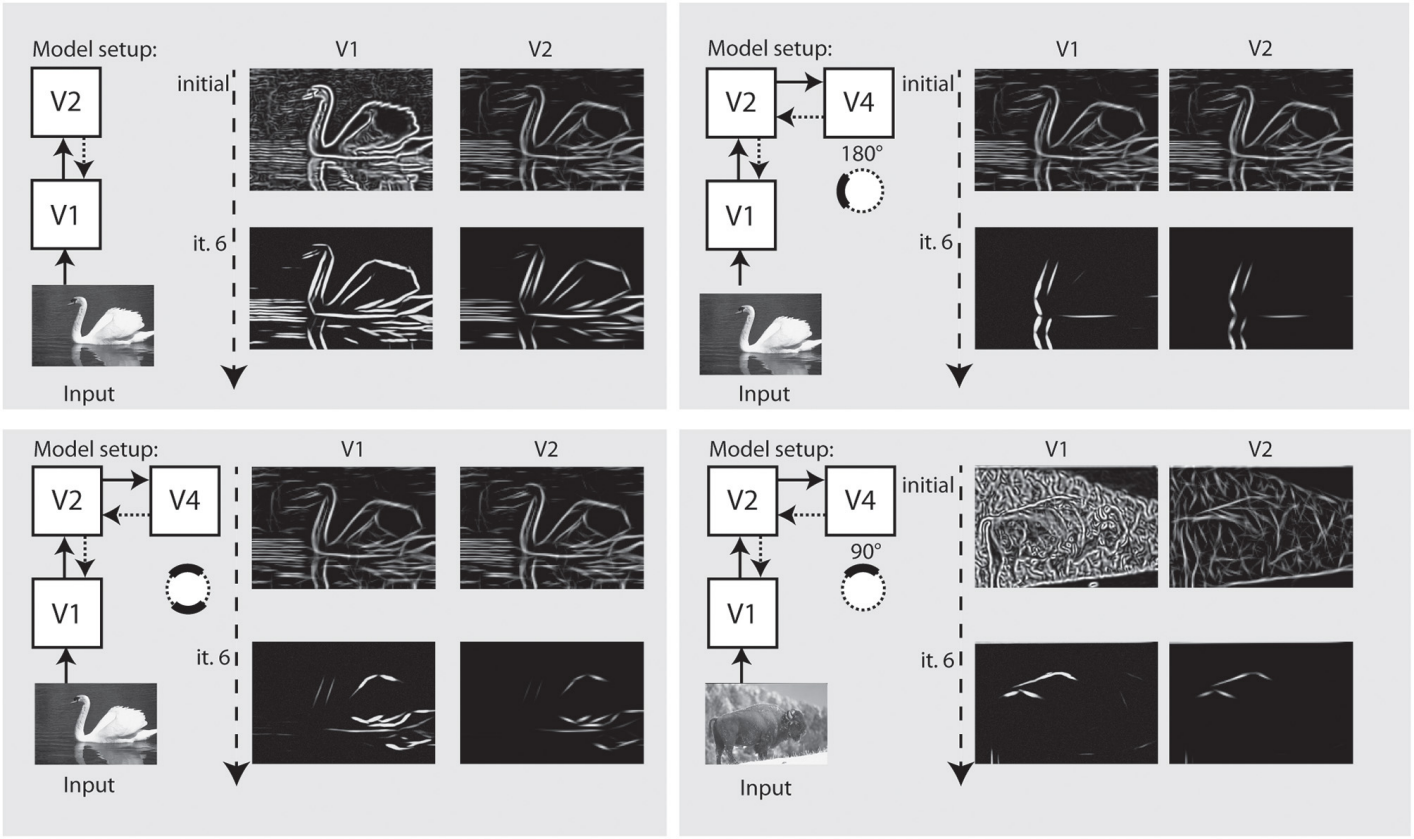
**Refinement and modulation of shape contours in a real world example. Left:** Within 5 iterations, the outline of the animal is very well visible at V1 and at V2 stage. **Right:** With modulatory feedback from model V4, various parts of the animal like those contours with a certain curvature and orientation can be emphasized.

In Figure 9 we perform the same selection for a realistic photograph depicting an elephant. On the *left* side, we show interaction of model V1–V2 causes an appealing representation of the animal at stages V1–V2. On the *right* side, we configured the model using model area V4 to emphasize parts of the outline of the animal that match a certain context and configuration, here, a part of the outline.

### 3.4. Border ownership and figure-ground assignment

**Figure 10:**
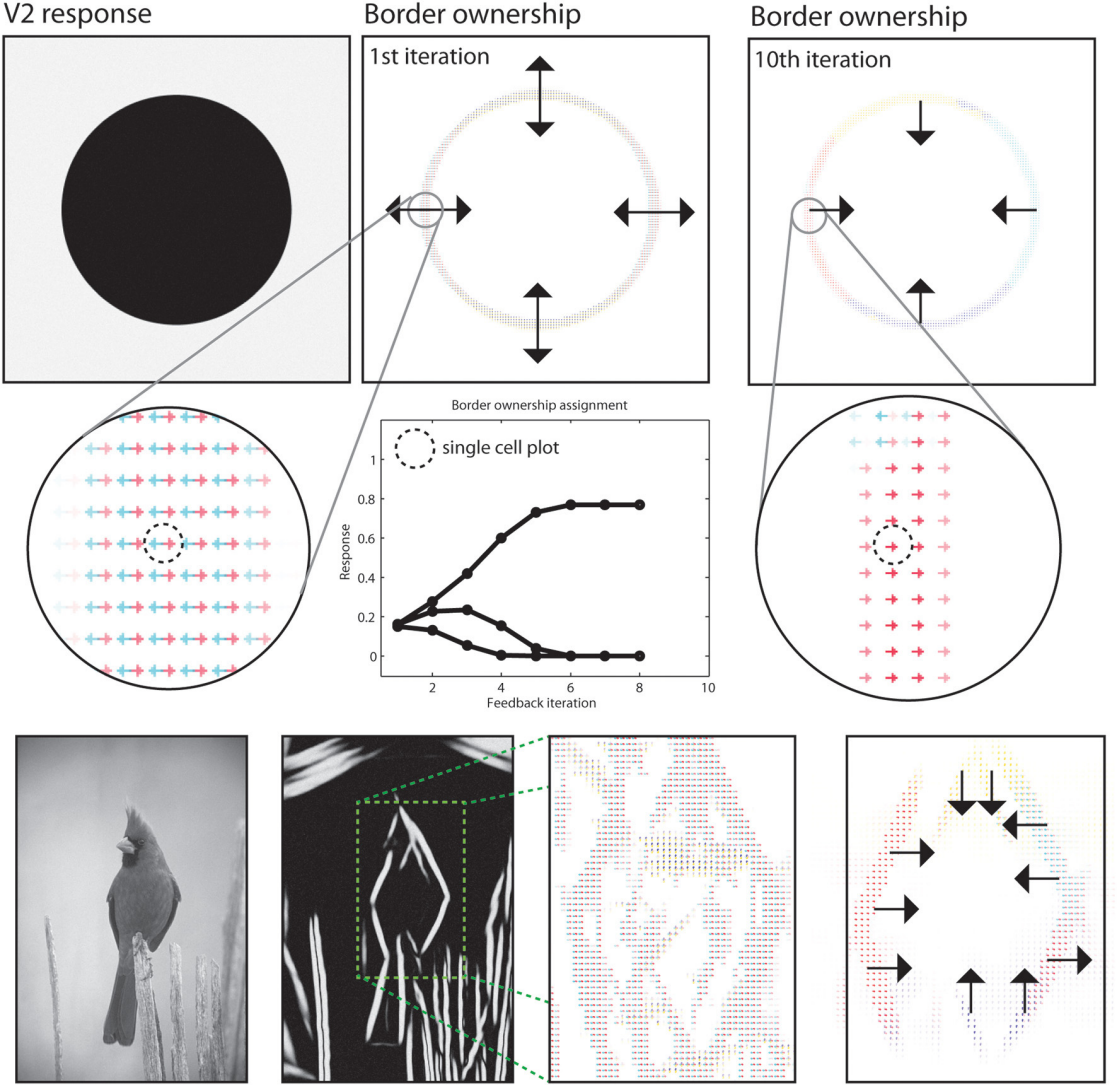
**Results for border ownership assignment**. First two rows: Cells at model V2 indicate the direction of figure side at positions where boundaries exist. Initially, four hypotheses exist for possible figure direction. These are refined in model V2, where only two hypotheses remain after the orientation of the boundary is represented. Contextual integration in model V4 then provides correct estimates with modulatory feedback. Subsequent normalization and mutual competition leaves only one hypotheses for border ownership direction. See text for details on the time phases of border ownership assignment. Third row: Demonstration of the boundary assignment for a natural image. The initial responses are improved after a few iteration steps.

In the segregation of a scene into figure and ground the modeled *border ownership* cells participate by indicating the direction where the frontal surface is positioned at a boundary (Zhou et al., 2000). Our model incorporates a mechanism using such border-ownership cells to resolve the direction of a frontal surface from local boundary cues (Zhou et al., 2000). We performed such a assignment for our sample images, see Figure 10 for an illustration of the result. The output of model area V1 and of V2 long-range integration cells are acquired to generate initial hypotheses of border ownership direction at image regions where local contrasts are situated. Initially, all four border ownership directions show equal responses at a boundary location. After stimulus onset, three dynamic effects occur and their contribution to the resolution of border ownership is reflected in the time course of cell activation, see Figure 10 for an illustration.

First, local feedback from V2 cells enhances two hypotheses of border ownership for the directions orthogonal to the local boundary orientations.

A local normalization causes an attenuation of the other two representations Figure 10, *second row*; Timestep 0 and 1). Second, shape-level integration at model area IT contributes positive feedback to those border ownership cells that are directed toward the inside of the figural depiction. Again, normalization leaves the net response of the cells constant (timesteps 2–4). Finally, mutual inhibition among border ownership cells with opposite direction selectivity causes the dominant direction to gain all available net energy (timestep 5–8). At this point, a stable point is reached and the local ambiguity for border ownership direction is resolved using feedback from higher cortical areas. The interpretation of the final representation would be that the frontal surface is to the inside of the curved boundary.

## 4. Discussion

### 4.1. Summary of contributions

In this contribution we emphasized the role hierarchical representations have in the organization of shape features and their combinations into a coherent form. Like some previous model developments (Cadieu et al., 2007; Hatori and Sakai, 2012; Rodríguez-Sánchez and Tsotsos, 2012) our model is based on low and intermediate representations of shape features. These proposals are all based on a strictly hierarchical feedforward processing sequence. We propose here that such shape encoding mechanisms may be based on distributed representations that are established by interacting assemblies each devoted to specific feature properties. Such interactions in the model are organized by recurrent interactions of feedforward and feedback signals. The underlying structural principles are based on the cortical architecture of the ventral pathway with mutual interactions between such distributed representations (Markov et al., 2013). The model architecture incorporates principles that have been predicted to minimize the computational efforts of visual systems to successfully deal with the complexity problem of perception (Tsotsos, 1988) [compare also (Tsotsos, 2005)]. Among those, the hierarchical organization of representations in model areas, the specific receptive field properties of model columnar mechanisms, hierarchical pooling of spatially separated input representations, and top-down (modulatory) feedback are proposed here to account for the functional properties of cortical shape processing. We did not discuss complexity advantages in this contribution. However, given the theoretical predictions by such earlier work our proposal of a model architecture provides a evidence how distributed intermediate-level mechanisms may help to shape our understanding of modeling complex visual machinery that captures key cortical principles.

The main contributions of the work presented in the manuscript are twofold. First, we propose a computational network architecture that utilizes a hierarchical distributed representation of shape features. Contour features play a major role to track moving shape in which their strength parametrically change as a function of their saliency (Caplovitz and Tse, 2007). This necessitates global configurational as well as local information to distinguish rather tiny differences in the outline of a 2-dimensional form [such as curved boundaries vs. localized corners (Pasupathy and Connor, 1999; Ito and Komatsu, 2004)]. In order to generate a representation with sufficient spatial resolution combined with spatial context we suggest that multiple specialized component representations interact by feedforward hierarchical processing that is combined with feedback from representations generated at higher stages in the hierarchy. Second, we incorporate grouping mechanisms to integrate like-oriented contour responses that are integrated if they form a smooth outline fragment of a surface boundary (e.g., Grossberg and Mingolla, 1985; Neumann and Sepp, 1999; Ben-Shahar and Zucker, 2004). Such grouping mechanisms operate at the stage of area V2 and are, thus, involved in the hierarchical processing of shape. Given the hierarchical processing and representation of boundary information in the ventral pathway (see the overview in Neumann et al., 2007) the shape processing observed in area V4 is mainly driven by the output of grouping responses. It may be supplemented by input from simple/complex cells in V1, a principle of convergent signal streams also used in the models described in Thielscher and Neumann (2003); Rodríguez-Sánchez and Tsotsos (2012). In addition, we suggest that the shape representation built at the stages of V4 and IT influences the assignment of border ownership in surface representation (Zhou et al., 2000) (see overview in Neumann et al., 2007). Model IT cells send modulatory feedback to those V4 cells that provide relevant input (in V4 and V2) such that the net sum of convex corners/curvatures determines the ownership direction. The proposed model thus combines separate findings about the generation of cortical shape representation with figure-ground segregation mechanisms by assigning border ownership.

### 4.2. Relation to previous models of shape representations in cortex

Visual shape recognition has already been investigated intensively by considering the 3-dimensional (3D) surface appearance for object recognition (Riesenhuber and Poggio, 1999; Serre et al., 2007; Mutch and Lowe, 2008; Yamane et al., 2008; Serre and Poggio, 2010) as well as 2-dimensional (2D) shape recognition (Schwartz et al., 1983; Mokhtarian and Mackworth, 1986; Mokhtarian, 1995; Rodríguez-Sánchez and Tsotsos, 2012). In the context of view-based models of object recognition stable views (Logothetis et al., 1995) are associated with 2D shapes so that their analysis can be considered as an intermediate stage of object processing (Cadieu et al., 2007). The computational model approaches of 2D shape representation can be subdivided into flat and hierarchical schemes. Examples of flat processing schemes, e.g., utilize Fourier descriptors (Schwartz et al., 1983), multi-scale representations of curvature features in the shape outline (Mokhtarian and Mackworth, 1986; Mokhtarian, 1995), or global schemes for integrating oriented line features (Wilson and Wilkinson, 1998). Hierarchical multi-layer processing schemes are based on different stages to generate an increasingly coarse-grained representation of shape features utilizing repetitive application of local filtering operations (Riesenhuber and Poggio, 1999; Cadieu et al., 2007; Rodríguez-Sánchez and Tsotsos, 2012). In order to resemble the feature selectivity of V4 cells in monkey cortex such cells build coarse-grained orientation-curvature representation of the shape under inspection. The hierarchical organization of a sequence of processing stages follows the idea of the Neocognitron (Fukushima, 1980, 1988) by developing low and intermediate representations of richer shape feature compositions (LeCun et al., 1998; Riesenhuber and Poggio, 1999; Mutch and Lowe, 2008; Tabernik et al., 2014). The orientation-curvature representation of V4 cells reported by Pasupathy and Connor (1999); Connor et al. (2007) has been investigated in the models reported in Cadieu et al. (2007); Rodríguez-Sánchez and Tsotsos (2012); Hatori and Sakai (2012). We share the principles of the hierarchical organization of processing and the emergence of rich orientation-curvature sensitivity in our proposal. Initial processing utilizes orientation sensitive filters to extract local oriented contrast. Unlike the previous models we incorporate a stage of boundary grouping at the interface between low and intermediate levels of representation. Such grouping operations integrate oriented contrast responses that are arranged in the local neighborhood of a target location. The local responses are enhanced by evaluating a support function that measures feature compatibility [(Neumann and Mingolla, 2001) for an overview and taxonomy of grouping schemes]. The measure of compatibility, or relatability, depends on the lateral integration that utilizes oriented weighting functions for contrast features arranged along a model shape outline, e.g., circular arcs with different radii (Parent and Zucker, 1989). Such a scheme thus implicitly incorporates curvature as a local contour feature. In order to make this explicit, different contour radii and signs of curvature (for individual orientations) have been considered in Rodríguez-Sánchez and Tsotsos (2012). Rather then implementing this curvature selectivity in a hard-wired scheme of local oriented filter conjunctions, we propose that this selectivity is generated via bottom-up and top-down filter mechanisms organized in a hierarchy. In this architecture the responses from model V2 contour groupings (based on different radii) are integrated by model V4 curvature sensitive cells with coarse bipartite odd-symmetric receptive fields (similar to simple cell profiles, but at much larger spatial scale). The sign of curvature is distinguished by cells of opposite polarity that mutually compete for each orientation. As a consequence responses are generated preferentially in cases where a single dominant curvature is present while responses are suppressed for straight contours which feed curvature cells symmetrically. The curvature radius is represented through a family of differently scaled integration sizes of such model V4 cells. Each of these cells have a specific peak selectivity. In the simulations we used three different sizes for each curvature sign. In order to make those cell responses selective to the feature specificity but mainly invariant to luminance contrast we suggested that each V4 cell response competes against the responses of other curvature selective cells in a local pool that interact via a mechanism of shunting inhibition. This leads to normalization of responses just like in those mechanisms proposed to account for various non-linearities at different stages in cortical processing, e.g., for context related contour responses in V1 (Carandini and Heeger, 1994; Carandini et al., 1999), attention selection (Carandini and Heeger, 2012), and higher level cognitive functions (Louie et al., 2011). Since the curvature sensitive model V4 cells, in turn, send feedback to their input contour representations in model V2 and filter response in model V1 those corresponding input activations will be enhanced. The amplitude of responses in distributed boundary representations will be amplified as an emergent net effect such that local salient curvature features in a shape outline will be amplified to yield distributed component feature representations of figural shapes.

These local boundary and curvature representations also feed mechanisms of border ownership assignment at the level of the model V4/IT complex. Such mechanisms have been investigated before in e.g. Zhou et al., (2000); O'Herron and von der Heydt (2011). Our computational framework belongs to the group of feedback models for border ownership encoding (see the overview of the current state in Williford and von der Heydt, 2013, see discussion below). We adopted this generic scheme by integrating responses from curvature selective cells with the compatible sign of curvature. In such a way the ownership configuration favors contributions from coarsely presented convex components. If a shape with multiple convex and concave segments is present then the ownership cells with opponent direction selectivities compete in order to arrive at a disambiguated assignment of surface belongingness. This makes the testable prediction that bumpy outlines should lead to slightly longer ownership disambiguation than for smooth convex shapes since the disambiguation will take more time when initially opposite assignment hypotheses coexist.

An additional investigation was argued to be of importance in the work proposed here. Several experimental investigations have reported that cells in extra-striate cortex selectively respond to corner junctions. For example, Ito and Komatsu (2004) (compare also Hegdé and Van Essen, 2000) reported that cells in area V2 selectively respond as to generate representations of sharp corners, or angles, selective for a particular opening angle. Similarly, Pasupathy and Connor (1999); Yau et al. (2013) show that area V4 cells respond to sharp shape corners with a sub-population of cells preferring sharp corners with different orientation and opening angles while another sub-population prefers smooth rounded corners. While the previous hierarchical models can account for the response selectivity for any of these generic corner types the perceptual representation of sharp localized features that allow, e.g., to distinguish between sharp and rounded corners remain unanswered. Sharp corners of any opening angle would be indistinguishable from the smooth variants of these corners given the increasing smoothing and subsampling of the visual representation while proceeding in the hierarchy. Our model argues in favor of a distributed representation: While shape sensitive cells at an intermediate level represent the salient shape protrusions (as in V4) the localized detail of an outline is represented at a higher spatial resolution in lower-level representations, e.g., in V1, V2, V3. In our model we suggest representations of smooth boundaries with different curvatures represented by groupings in model V2 while sharp corners are implicitly represented by convergent V1 input in local representations in model V2/V3. We assume that responses of cells in the model V2/V3 complex mutually compete such that their energy provides a measure to normalize individual responses. These provide convergent input to curvature selective contour cells in model V4 which, in turn, send feedback signals to their input sites at preceding stages. Since they are driven by either smooth or sharp contour arrangements the interaction of bottom-up sensory and top-down context-driven signals leads to selective enhancement of the particular corner configuration in the present stimulus. The specific details of the interaction between such counter-stream signal flows are discussed below.

### 4.3. Feedback as prediction mechanism to link shape components

The hierarchical model architecture proposed here is composed of multiple model areas each of which is represented by a three-stage columnar cascade model. In a nutshell, the model cascade consists of (i) an initial stage of input filtering, (ii) a stage of activity modulation of filter outputs by top-down or lateral re-entrant signals, and (iii) a stage of center-surround interaction of target cells against an inhibitory pool of cells leading to activity normalization to generate the net output response of the model area. These three stages can be roughly mapped onto compartments of cortical area subdivisions (as suggested in Self et al., 2012). The filtering stage of the driving feedforward input signals is specific to the particular (model) area under consideration. At the output stage, the activity normalization is computed by a mechanism of shunting inhibition, like the non-linear divisive mechanisms proposed in Carandini and Heeger (1994); Carandini et al. (1999); Kouh and Poggio (2008); Carandini and Heeger (2012). The feedback signal is generated at higher-level cortical stages or parallel processing pathways and is thought to provide context information that is re-entered at the stage earlier in the processing hierarchy (Grossberg, 1980; Edelman, 1993).

The functional role feedback signals play still remains controversial. Different proposals how feedback signals interact and combine with the driving feedforward stream have been discussed in the literature which have received different support from the experimental literature (Markov et al., 2013). One such framework proposes that the goal of computation is to reduce the residual error between the different signal streams in order to approach the sensory prediction generated by higher stages of processing (Ullman, 1995; Bastos et al., 2012). This idea is rooted in the Bayesian theory of predictor-corrector mechanisms which yields to the Kalman optimal filter realization under some restricting assumptions (Rao and Ballard, 1999). We follow an alternative route in which the feedback mechanism is modulatory in nature. Unlike predictive coding which tried to drive the difference between driving signals and the prediction to zero bottom-up input signals are amplified by matching feedback signals. This leads to a gain enhancement for those cell responses where a matching top-down predictive signal template has been generated. This feedback signal amplifies the sensory signal such that the subsequent competition between neurons yields a competitive advantage for the enhanced response patterns [biased competition; (Girard and Bullier, 1989; Desimone, 1998; Roelfsema et al., 2002; Reynolds and Heeger, 2009)]. The modulation mechanism is reminiscent of the linking mechanism suggested by Eckhorn et al. (1990); Eckhorn (1999) to account for activity synchronization in networks of spiking neurons. We have recently demonstrated (Brosch and Neumann, 2014) that such mechanism of convergent bottom-up feedforward and top-down feedback signal correlation accounts for the signal amplification as measured at the level of cortical pyramidal cells (Larkum, 2013).

In the shape processing architecture described here the modulatory feedback serves the role of a predictor (Spratling, 2008). For example, bottom-up input in oriented contrast is integrated by mechanisms of contour grouping and integration to generate continuous boundary representations. This is similar in spirit as the recent investigation of Piëch et al. (2013) who emphasized how context information at higher cortical stages influence more local feature representation at lower levels. Here, the same principle is replicated over different stages of model cortical processing. Contour representations after grouping in model V2 and junction configurations in model V3 send their output activations to curvature sensitive cells in model V4 where the activities are integrated. These cells, in turn, send their feedback to the input populations of neurons that have generated their input. The computational logic is that the curvature responses provide a template of context-related information about the local presence of oriented shape features. The modulatory feedback amplifies those inputs that are consistent with the curvature feature representation. The mutual competition of responses in a pool of cells at the lower level leads to a suppression of inputs that do not contribute to the present curvature feature. In all, a distributed representation of shape information is created that contains coarse-grained configurational information about stimulus shape and, at the same time, the spatially localized detail needed to distinguish between sharp and smooth corners. Similarly, the action of feedback sent from ownership sensitive cells (in the V4/IT complex of the model) to curvature sensitive and grouping cells in model V2 and V4 also provides context information for the assignment of configurational information. Here, the ownership assignment is based on the consolidation of evidence which convex shape elements make to establish a closed shape region in the visual field. This context is delivered via feedback to their input that represents fragments of shape components (irrespective of the sign of curvature) and also to the grouping representations. Those shapes that finally receive assigned direction of border ownership, and thus figure-ground direction, will enhance the associated inputs at the intermediate level orientation-curvature representations.

In all, the hierarchical processing scheme proposed here relies on extensive bidirectional flow of information in which the feedback signals that represent context-sensitive templates are gated by feedforward driving input signals. Such a modulating feedback driven gain control mechanism relates to mechanisms proposed by Roelfsema and colleagues (Lamme and Roelfsema, 2000; Roelfsema et al., 2002; Roelfsema, 2006) in which spatial detail is generated by feature-driven low-level processes and representations and subsequently associated with coarse-grained context information provided by intermediate and higher-levels of cortical computation. The mechanisms implemented in the proposed model are consistent with theoretical predictions from computational constraints visual perception imposes on the underlying architecture (Tsotsos, 1988). The advantages in computational complexity have been calculated for principles such as hierarchical organization, localized receptive field computations, and dedicated (distributed) maps of feature representation and their combination. Feedback has been suggested to steer an attentional beam by selecting a spatial region and their computational resources (Tsotsos, 2005). In the proposed architecture feedback also selectively enhances representations of features by increasing their gain which are coherent with the predictions generated at higher-level stages with more condensed coding of shape and figural properties. Also we emphasize that this provides a key to enhance (and make accessible) localized shape features, such as sharp edges, as part of a shape configuration that is represented on a coarser scale.

### 4.4. Model limitations and further extensions

The proposed model architecture emphasized the computational role of feedforward and feedback mechanisms in order to generate a hierarchical distributed representation of shape information. For that reason, we focused on the representational aspects as steady-state solutions of an otherwise dynamic interaction between neuronal populations and representations distributed over several model areas. We did not, so far, investigate the temporal response phases observed for shape sensitive cells in V4 (Yau et al., 2013). The work of Roelfsema and colleagues has shown that different response phases exist that can be reliably assigned to different mechanisms in processing, namely for feature detection, figure-ground segregation, and attention (Roelfsema et al., 2007). We have demonstrated that such separate but temporally overlapping phases can be accounted for by a recurrent network of mutually interacting neuronal sites. The network model has been composed of the same components like the present model architecture (Raudies and Neumann, 2010). It would thus be interesting to reveal whether similar temporal phases can be identified for model V4 cells that may give rise to identify different signatures indicative of contributions from delayed neuronal mechanisms that are involved in the computation of figural shape information.

Different signal streams (particularly in the feedforward sweep of feature processing) operate on different temporal scales. Several lines of evidence suggest that the dorsal and the ventral streams of processing do not operate entirely in isolation but mutually interact at different levels (Felleman and Van Essen, 1991; Markov et al., 2013). Also different response characteristics of cells may define different temporal routes of fast and slow processing (Born, 2001) that may help fusing information from different pathways. Here, we did not take into account such interactions based on different temporal effectivenesses. However, other model investigations capitalized on combining information from different channels to improve the selectivity of representation. For example, edge detection and grouping (in the ventral pathway) could be enhanced through mutually inhibitory gain control (which is similar as the normalization stage described here) generated by representations in the dorsal pathway. Since the dorsal representation is created by magno-cellular responses, such inhibition arrives already early to shape the selectivity of shape representations in the ventral path that is mainly driven by parvo-cellular responses (Shi et al., 2013). Similarly, interactions between the motion and form pathway have been suggested to help disambiguating localized features that give rise to occlusion cues which, in turn, support the disambiguation of object representation in the motion representation (Bayerl and Neumann, 2007; Beck and Neumann, 2010). Such detailed mechanisms would further enhance the proposed model architecture in refining the selectivities at different levels of low and intermediate representation.

As already pointed out above, the focus here is on the processing of 2D shape representations. In Cadieu et al. (2007) the authors have highlighted that their specific model investigation on shape representation in V4 is part of a larger hierarchically organized architecture for object recognition (Riesenhuber and Poggio, 1999; Serre and Poggio, 2010). Since their model principles relied on purely feedforward processing the insights provided in the work presented here might also shed some light on the mutual interactions between different processes on an even larger scale of object recognition processes. In addition, it would be interesting to find out how the representation of 3D surface patches (Yamane et al., 2008) seamlessly fit into a model computational architecture of recurrent shape computation.

In the presented coverage our model does not respond to contours elicited by contrasts of spatial luminance statistics caused by differently textured regions. However, the core mechanisms, including initial filtering, modulatory feedback and competitive interaction for normalization, are like those proposed in the current contribution. A model that focuses on the processing of such boundaries has been developed in Thielscher and Neumann (2003). It is thus very likely that the recent model architecture proposed here can be extended with processing stages capable to process texture define boundaries as well without changing the basic architecture and computational principles. Also not considered in the current version is a multi-scale approach. We acknowledge the theoretical justification of hierarchical multi-stage processing to build up a pyramid-like structure (Tsotsos, 2005). Incorporating this representational diversity would allow the processing of a wider range of curvature configurations in shape outlines. In addition, this would support a more robust segregation of border ownership on the basis of convexities in the figural outline. We have focused our efforts on the specification of a hierarchically organized network architecture that utilized bottom-up and top-down convergent processing flows. In order to keep the computational efforts and the simulation times within reasonable bounds we restricted our description to single scale components at the different model stages within the hierarchy. A more extended realization of components is certainly desired but left for future investigations.

Intermediate level representations involve cells with receptive fields that recruit multiple sub-field components (Mineault et al., 2012; Yau et al., 2013). The model of Cadieu et al. (2007) accounts for this by sequentially fitting the subunits of intermediate level receptive field models to match the response profiles of V4 responses measured experimentally. This yields a sampling structure of statistically significant inputs in a feature space that contributes a significant amount of feature input to generate the final response of a shape selective cell. So far, in our modeling we sampled the spatial and the feature domains regularly. This of course demands high representational as well as computational resources. Consequently, it would be of interest to see how an irregularly sampled 4D space-feature domain (with orientation and curvature features) can be embedded into the scheme of shape representation proposed here.

### Conflict of interest statement

The authors declare that the research was conducted in the absence of any commercial or financial relationships that could be construed as a potential conflict of interest.
